# Survey and analysis of microsatellites from transcript sequences in *Phytophthora *species: frequency, distribution, and potential as markers for the genus

**DOI:** 10.1186/1471-2164-7-245

**Published:** 2006-09-28

**Authors:** Diana P Garnica, Andrés M Pinzón, Lina M Quesada-Ocampo, Adriana J Bernal, Emiliano Barreto, Niklaus J Grünwald, Silvia Restrepo

**Affiliations:** 1 Laboratorio de Micología y Fitopatología Uniandes (LAMFU), Universidad de Los Andes. Bogotá, Colombia; 2 Centro de Bioinformática-Instituto de Biotecnología (IBUN), Universidad Nacional de Colombia. Bogotá, Colombia; 3 Horticultural Crops Research Laboratory, USDA ARS, Corvallis, OR, 97330, USA

## Abstract

**Background:**

Members of the genus *Phytophthora *are notorious pathogens with world-wide distribution. The most devastating species include *P. infestans, P. ramorum *and *P. sojae*. In order to develop molecular methods for routinely characterizing their populations and to gain a better insight into the organization and evolution of their genomes, we used an *in silico *approach to survey and compare simple sequence repeats (SSRs) in transcript sequences from these three species. We compared the occurrence, relative abundance, relative density and cross-species transferability of the SSRs in these oomycetes.

**Results:**

The number of SSRs in oomycetes transcribed sequences is low and long SSRs are rare. The *in silico *transferability of SSRs among the *Phytophthora *species was analyzed for all sets generated, and primers were selected on the basis of similarity as possible candidates for transferability to other *Phytophthora *species. Sequences encoding putative pathogenicity factors from all three *Phytophthora *species were also surveyed for presence of SSRs. However, no correlation between gene function and SSR abundance was observed. The SSR survey results, and the primer pairs designed for all SSRs from the three species, were deposited in a public database.

**Conclusion:**

In all cases the most common SSRs were trinucleotide repeat units with low repeat numbers. A proportion (7.5%) of primers could be transferred with 90% similarity between at least two species of *Phytophthora*. This information represents a valuable source of molecular markers for use in population genetics, genetic mapping and strain fingerprinting studies of oomycetes, and illustrates how genomic databases can be exploited to generate data-mining filters for SSRs before experimental validation.

## Background

*Phytophthora *spp. are notorious, world-wide pathogens because of their devastating effects on many crop species, that often result in significant economic losses. All members of the genus *Phytophthora *infect plants, although some exhibit a broad host range and others infect only a few species [1]. Among the most important species are *P. infestans, P. sojae *and the newly described species, *P. ramorum *[2]. *P. infestans *affects several host plants from the Solanaceae family including potato, tomato and a number of tropical fruits economically important for producer countries [3,4]. Current losses in potato production worldwide reach $5 billion [5]. The soybean pathogen, *P. sojae*, causes root rot and damping off, and in the US alone has recently accounted for annual losses in the order of $1–2 million [1]. *P*. *ramorum *has only been described relatively recently, so that basic information about its biology and ecology is scant. This pathogen causes sudden oak death disease and is currently decimating trees and shrubs in the coastal oak forest in California, including keystone tanoak and coast live oak species, and might be expanding to other hosts such as redwoods and to other regions in North America [6].

Given the economic relevance of these pathogens, it is important to standardize high-throughput molecular methods for routinely characterizing *Phytophthora *populations. Genomic and bioinformatics resources have grown exponentially in recent years, generating information more rapidly than current data-processing tools can handle. Thus, the generation of new tools and the application of existing ones for exploring databases constitute a practical and inexpensive approach to elucidating biological systems. The application of bioinformatic tools to genomic databases that are already available for different species of *Phytophthora *could allow techniques for characterizing these pathogens to be developed rapidly.

Two of the most informative databases available for *Phytophthora *are the EST (Expressed Sequence Tags) public databases and the Department of Energy's Joint Genome Institute (JGI) databases in which complete genomes, predicted proteins and predicted transcripts are available for *P. sojae *and *P. ramorum *[7]. Transcript sequences constitute a rich and special source of informative molecular markers because they represent genes that are expressed in an organism. Coding sequences are generally more informative than anonymous markers because they allow for a more direct association between the molecular marker and the phenotype. Microsatellites or simple sequence repeats (SSRs) are molecular markers that consist of tandem repeats of one to six DNA base pairs. SSRs are highly versatile, PCR-based markers, usually associated with a high frequency of length polymorphism [8]. They are choice markers given that they show fairly high mutation rates and are codominant [9,10]. They have been found in both coding and non-coding DNA sequences of all higher organisms analyzed [11,12]. In oomycetes, SSRs have had important applications such as diagnosis and determination of mating type [13], genetic structure and disease dynamics [14,15], and population genetics [16,17]. Apart from their application as molecular markers, determining the abundance and density of SSRs in oomycetes may help understand whether these sequences have any functional and evolutionary significance [18].

An innovative marker system that has been developed links expressed sequence tags (ESTs) and SSRs [19,20]. These EST-SSRs have been applied successfully in studies of genetic variation, linkage mapping, gene tagging, evolution and sequencing of several plant genomes [21]. Although they are less polymorphic than genomic SSRs, EST-SSRs tend to be more conserved, at least in the plant species in which they have been studied [22]. This characteristic makes EST-SSRs readily transferable between related organisms [17,23-26]. Therefore, SSRs from transcript sequences have considerable potential for comparative mapping studies, as well as for analyses of genetic diversity within the expressed portions of the genome in which they are located.

Although *Phytophthora *EST and transcript databases are publicly available, no formal analysis of SSRs in these sequences has been reported. We used an *in silico *approach to analyze the frequency and distribution of SSRs in transcript sequences from the oomycetes *Phytophthora infestans*, *P. sojae *and *P. ramorum*. Previous studies on oomycete phylogeny have suggested that these three species are monophyletic and that *P. sojae *and *P. ramorum *are more closely related to each other than to *P. infestans *[27-29]. We also surveyed the distribution and possible patterns of SSRs in selected sequences corresponding to genes previously associated with pathogenesis and virulence. In addition, we studied *in silico *the transferability of these SSR-based markers between species. We generated primer pairs, where possible, for all SSRs from these three organisms. A publicly available database was generated for *Phytophthora *microsatellites, including primers and SSR survey results [30]. This study will serve as reference for future comparative mapping studies and for the development of strategies that take advantage of DNA sequence analyses for cross-referencing genes between species and perhaps genera.

## Results

### Microsatellites: motif, length and frequency

Consensus EST databases from *P. infestans *and annotated transcripts from *P. sojae *and *P. ramorum*, were scanned for the presence of microsatellites, defined as short tandem repeat motifs of 1–6 bp. We also explored the existence of 7–10 bp motifs, which represent the transition between micro and minisatellites; little is known about these. A total of 84000 available EST sequences were downloaded from the *Phytophthora *Functional Genomics Database [31] for *P. infestans*, and 19276 and 16066 predicted and annotated transcripts were downloaded from the Department of Energy's Joint Genome Institute [32]. The EST set was masked for repetitive sequences, obtained from RepBase ([33]; which does not include SSR repeats), assembled and revised manually to generate consensus sequences. We used consensus EST sequences because they have the built-in advantage of eliminating redundant SSR counts, allowing us to make more precise estimates of SSR frequency. The assembly process resulted in 25965 sequences for *P. infestans*. Annotated transcripts were not subjected to further treatment, and along with the consensus ESTs were used for the SSR survey.

The frequency of repeat motifs in the consensus EST sequences and annotated transcripts was assessed. A first analysis was performed with only the consensus ESTs from *P. infestans*; more than 50% of the SSRs identified were (A)_n _, which is an over-representation with respect to the other mononucleotide repeats as well as to other motifs. Although mononucleotide repeats are common in genomic DNA and can be valid SSRs, most of those present in expressed sequences are the result of nucleotide additions by RNA polymerase and are not present in the genomic DNA template (e.g. poly-A tails; [34]). Therefore, the analysis of monomers was excluded and only motifs with repeats of 2 to 6 bp (for ESTs and transcripts) were included in this study. Both perfect and compound SSRs were selected with a minimum acceptable length of 12 bp for di, tri and tetra-nucleotide motifs. Only SSRs with a minimum of three repeats were included in the analyses of penta- and hexa-nucleotide repeats. The total counts, frequencies and comparisons of SSRs (2–6 bp) in each set of sequences are summarized in Tables 1, 2, 3.

*P. infestans *had the most sequences analyzed but showed the lowest total SSR count and percentage of SSR-containing sequences (Table 1). However, this result has to be considered wih caution since the total size (Mb) of the sequences examined for *P. infestans *is approximaely half that of the other organisms. To compare the organisms more realistically, another approach was required: namely, taking the total length of each set of sequences analyzed as a reference. Thus, total relative abundance and total relative density were calculated (Table 1). Statistically significant differences (P < 0.05) were found between *P. infestans *and *P. sojae *and between *P. sojae *and *P. ramorum *when we compared the total sequence lengths (bp) contributed by SSRs with respect to the total megabases of examined sequences (relative density), but there was no difference between *P. infestans *and *P. ramorum*. The same differences were found when perfect and compound SSR proportions were compared (data not shown), demonstrating that SSR content is not in agreement with the phylogenetic distances between these organisms [6]. The numbers of SSR-containing sequences and sequences containing more than one SSR were compared only between *P. sojae *and *P. ramorum *because of the different types of source sequences (ESTs for *P. infestans *and transcripts for *P. sojae *and *P. ramorum*). All comparisons showed statistically significant differences (data not shown). These results might imply that the net SSR content in transcript regions of *Phytophthora *species could be variable, and again, not directly related to phylogenetic distance.

**Figure 1:**
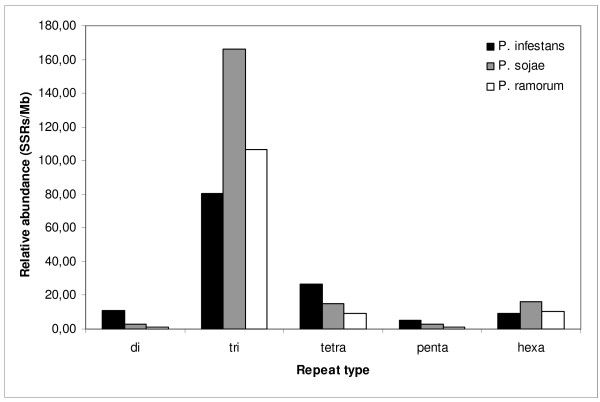
**Relative abundance of SSRs among oomycete consensus ESTs and transcript sequence sets in the oomycetes analyzed**. Abundance is defined as the total number of SSRs per Mb of sequence analyzed.

**Figure 2:**
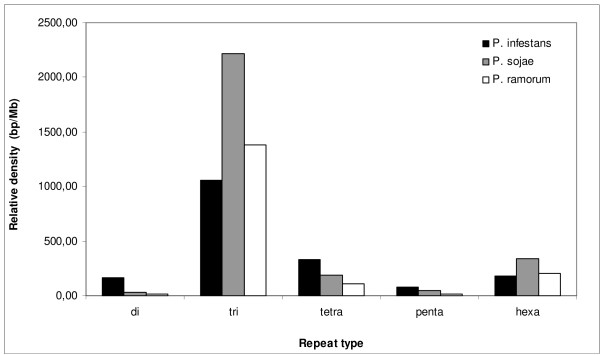
**Relative density of SSRs across consensus oomycete ESTs and transcript sequence sets**. Density is defined as the total sequence length (bp) contributed by each SSR per Mb of sequence analyzed.

The total numbers of all types of microsatellite motifs are shown in Table 2 and Figures 1 and 2. All three sequence sets contained SSRs that were mainly trinucleotide repeats (> 60%), while the dinucleotide repeats represented less than 8%. This agrees with results from other eukaryotes, where trinucleotide repeats are overrepresented in coding sequences in comparison with dinucleotide repeats [35,36]. Hexanucleotide repeats constituted the second most frequent motif in *P. sojae *and *P. ramorum*, differing markedly from *P. infestans*, where this motif has one of the lowest percentages. In general, statistically significant differences were not found between *P. sojae *and *P. ramorum *(proportions test, P > 0.05), showing that in contrast to the net SSR content, SSR distributions could be related to phylogenetic distances among *Phytophthora *species. Relative abundance and relative density allowed the similarities and differences among SSR distributions to be represented graphically (Figures 1 and 2). The high relative abundance and density values observed for tri- and hexa-nucleotides might correlate with coding region stability ([37]; see discussion).

The fifteen most frequent motifs were analyzed for all three organisms in terms of percentage and total counts (Table 3). Most of the motif types were present, and the five most frequent motifs were the same, in all three *Phytophthora *species; however, more motifs were shared between *P. sojae *and *P. ramorum *(~80%) than between these two species and *P. infestans *(~46%). The most common trinucleotide repeats in all cases were (AGC/CGT), (ACG/CTG) and (AAG/CTT); the most common dinucleotide repeat for *P. sojae *and *P. ramorum *was (CG/GC), in complete contrast to *P. infestans*, where this motif was least frequent. Tetra-, penta- and hexa-nucleotide repeat motifs showed no clear trend among the three organisms. In general, these results and those from the SSR distributions, reflect the more close relation between *P. sojae *and *P. ramorum *at the sequence level.

Since the most common motifs in the three organisms analyzed were trinucleotide repeats, we attempted to identify the amino acid(s) encoded by these. Twenty sequences from each organism, containing the most common triplet in each case, were randomly selected and used for ORF analyses. The most probable open reading frame and consequently the location of the SSR in this reading frame were determined. The triplets analyzed (in their canonical forms and possible variations) were (AGC)_n _for *P. sojae *and *P. ramorum *and (AAG)_n _for *P. infestans *respectively. In *P. sojae *and *P. ramorum*, CAG (glutamine) was predominant at 80% and 60% respectively, followed by AGC (serine) at 10% and 35%, and finally GCA (alanine) at 10% and 5%. In *P. infestans*, GAG (glutamic acid) was predominant at 50%, followed by AAG (lysine) at 30% and finally CTT (leucine) at 10%. These results were further investigated for correlations between the amino acids encoded by the trinucleotide repetitions and the codon usage preferences reported for each organism in the Codon Usage Database from the Kazuka DNA Research Institute [38]. In all cases, the triplets analyzed encoded for amino acids that were normally overrepresented in the corresponding organism. The differences detected between *P. infestans *and *P. sojae-P.ramorum *emerging in our trinucleotide analysis were also corroborated by codon usage frequencies.

**Figure 3:**
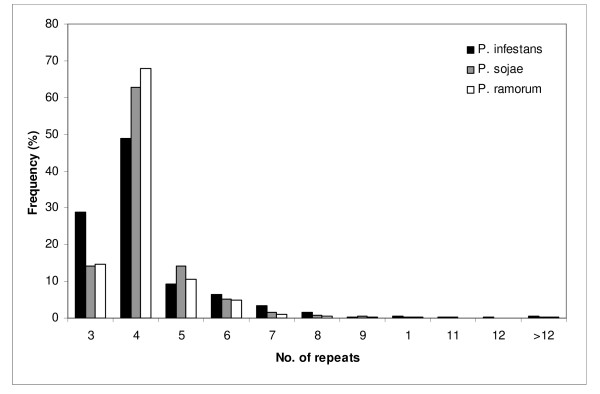
Frequency of repeat unit numbers of SSRs from *P. infestans *(black),*P. sojae *(grey) and *P. ramorum *(white).

Repeats containing motifs between 7 and 14 bp were scarce in the three oomycetes transcriptome analyzed, accounting for less than 1% of the total number of SSRs. They did not merit further attention in our study because of their low abundance. Regarding SSR lengths, we found that di-, tetra- and penta-nucleotide motif types did not exceed 30 bp (Table 4), while tri and hexanucleotides were clearly longer in all organisms. Although *P. sojae *had the longest SSR for di-, tetra-, penta- and hexa-nucleotide repeat types, longer than reported for other organisms (Karaoglu *et al*., 2004), only a very few of these lengths can be considered "long microsatellites" (> 15 repetitions [39]). The motif sequences of the longest SSRs are not shared among organisms, indicating that this factor is independent in each species. Thus, our results suggest that long SSRs are absent from these consensus EST or transcripts sequences. The number of repeats found in SSR loci ranged from 3 to 33, from 3 to 32 and from 3 to 15 in *P. infestans, P. sojae *and *P. ramorum*, respectively. Most SSR loci showed seven repeats or less (96% *P. infestans*, 98% *P. sojae *and 99%*P. ramorum*), with a repeat number of four being the most common in all species (Fig. 3).

### SSRs in pathogenicity factors

In total, 136, 318 and 171 sequences corresponding to pathogenicity factors or annotated as putatively involved in pathogenicity were selected for *P. infestans, P. sojae *and *P. ramorum*, respectively, and their SSR distributions were characterized. They included enzymes such as cell wall degrading enzymes (cutinases, glucanses, polygalacturonases, pectate lyases and cellulases), elicitins and avirulence homolog proteins with conserved RXLR motifs, and other secreted proteins potentially related to pathogenicity [7,40]. SSRs were also surveyed in two additional sequence sets corresponding to constitutively expressed genes: ribosomal and housekeeping genes such as actin, cytochrome P450-like protein and NADH hydrogenase. The results showed that the percentage of SSR-containing sequences in the pathogenicity factors was not significantly different from that in the ribosomal and housekeeping genes (Table 5). The scarcity of SSRs in the putative pathogenicity factors suggested that SSR length variation has no significant influence on the mutation rate in these sequences.

### Primer design for EST-SSRs and databases

Sequences flanking microsatellites from each of the three organisms were used to develop primer pairs using Primer3 software [41]. For this first survey, the parameters were not stringent since a high number of sequences were analyzed. We produced primer pairs for 61.44%, 87.47% and 88.97% of the SSRs from *P. infestans, P. sojae *and *P. ramorum *respectively; not all the SSRs were located in positions suitable for optimum design. Three different primer pairs were generated for each SSR and deposited in the developed database [30] with their sequences, the consensus EST or transcript sequence (with the original sequence ID) and a brief description of the amplifiable SSR. These primers can be used to amplify the corresponding SSR region for diverse applications, so they constitute a publicly available resource for future research. Of the three primer pairs, only the first (the best score) was used to analyze transferability.

### *in silico *analysis of transferability

Primer pairs for each SSR locus were assayed *in silico *for cross-transferability. Primers for each organism were aligned against the consensus ESTs or transcript databases from the other two organisms. Three criteria were established to filter the comparisons: (i) high degree of similarity (> 90%) between primer and aligned sequence; (ii) primers aligning with only one sequence containing an SSR; (iii) a hypothetical PCR product size longer than 100 bp. Of all the primers designed (8739), 7.5% appeared transferable between at least two species. Not surprisingly, most of the virtually transferable primers were found between *P. sojae *and *P. ramorum*, since these are the two most closely related species. However, 84 (~1 %) primers were found to be virtually transferable among all three species (Table 6).

## Discussion

The present study was designed to create microsatellite databases for *P. infestans*, *P. sojae *and *P. ramorum*, taking advantage of publicly available sequences for these organisms. In the case of *P. infestans*, EST sequences were first assembled and then explored for SSRs. For *P. ramorum *and *P. sojae*, annotated transcripts from the available whole genome sequences were used directly to search for SSRs and to analyze the distribution and organization of SSRs in the transcribed regions of these organisms. Approximately 6–22% of the sequences contained SSRs, which shows higher frequencies than previously reported for plant ESTs [25,37,42], fungal endophytes [43] or other higher eukaryotes [8,44]. However, these differences might reflect the different criteria used to select the SSRs. Repeat sequences of at least 12 bp for di-, tri- and tetra-nucleotides, and three or more repeated units for penta- and hexa-nucleotides, were chosen for this study. These lengths were used because they have been considered the minimum acceptable microsatellite lengths [34] and are efficient thresholds for detecting high levels of polymorphism [22]. Our results showed that SSR lengths are very restricted in the coding regions of oomycete genomes; approximately 99% of all the SSRs analyzed were shorter than 30 bp. Only a few SSRs had higher numbers of repeat units, perhaps because of their location in well-conserved regions of the genome. Strong evolutionary and functional constraints limit the expansion of microsatellite repeats in expressed regions of the genome [45,46], because longer repeats have higher mutation rates and could therefore be less stable [47,48]. Short microsatellites are probably generated by random mutations and then expanded by DNA polymerase slippage. Thus, the base composition of a sequence that seeds the evolution of repeats is expected to influence microsatellite density [49,50]. Therefore, the similarities between SSR motifs within oomycetes and between oomycetes and fungi may indicate that specific common sequence composition contributes to the evolution of SSRs.

Statistically, there were no quantitative differences in the distributions of SSR motifs between *P. sojae *and *P. ramorum*. However, marked differences were found when these two species were compared with *P. infestans*. This is particularly interesting because the frequency distribution of SSRs does not follow a genus-wide pattern; if it is strongly species-dependent, it could indicate evolutionary events specific to the organisms compared. In addition, SSRs derived from exonic regions, which are more conserved than genomic SSRs, might consequently show only minor differences in distribution among related species. Thus, differences in genomic organization could explain the SSR distributions observed in the organisms examined, reflecting the phylogenetic distances between them [29,51]. More sequence data on ESTs/transcripts will soon become available for Stramenopiles, as *P. infestans, P. capsici, Pythium ultimum*, and *Hyaloperonospora parasitica *are or will be sequenced in the near future that will provide further resolution on the evolution of SSR motifs in Oomycete coding regions.

The most common SSRs comprised trinucleotide repeat units with low repeat numbers. A wide variety of repeat motifs were represented at high percentages in these trinucleotide arrays. The abundance of repeat motifs differed slightly, especially between *P. infestans *and the other two species; (AGC)*n*, (ACG)*n *and (AGG)*n *were the most abundant triplets in all organisms but their abundances differed among species. This finding was expected, since EST or transcript-derived microsatellites are likely to be conserved in frequency, abundance and distribution across closely-related species [52]. A database search of all possible trinucleotide repeat motifs (>20 bp) showed that (AGG)*n*, (AAT)*n *and (ATC)*n *are relatively common in fungi, but (ACG)*n *and (CCG)*n *are relatively rare [53]. Differences in abundance and density among trimeric repeats could be explained by species-specific cellular factors that interact with the motifs and play an important role in generating the repeats [18]. Among plant species, the abundances of different repeat motifs in EST-derived SSRs vary greatly. However, trinucleotide units with low repeat numbers are common features of EST-SSRs [25,37,42,54]. Our results suggest that *Phytophthora *spp. might have a set of common motifs, as is the case with fungi, whereas the motifs in oomycetes may vary widely, as they do in plants.

High dinucleotide repeat abundances in whole genomes have been reported for fungi, *Drosophila*,*Caenorhabditis elegans *and a subset of plant genomes [36,51,54,55]. Dinucleotide repeats have been characterized as being the most important SSRs because of their higher mutation rates [51]. This suggests an explanation for their high abundance in genomic regions and low abundance in coding regions, which must be conserved to maintain functionality. On the other hand, many studies have reported that trinucleotides are most abundant in coding regions of higher eukaryotic genomes [46,56,57]. Previous studies have shown that trinucleotide repeats predominate in plant EST libraries, supporting our observations on oomycetes [12,58]. Among all SSRs, expansions or deletions in coding regions can be tolerated for tri- and hexa-nucleotides, which do not perturb reading frames [36]. This could explain why the longest SSRs in all the organisms examined belong to these motif categories, although the three-dimensional structure of a protein translated from a sequence containing such SSRs would not necessarily be unchanged. Interestingly, our analyses showed that glutamine is the amino acid encoded by the most highly abundant repeat in *P. sojae *and *P. ramorum*: (CAG)_n _. This agrees with previous reports on *Drosophila*, *C. elegans *and yeast [36], in which the same glutamine-coding triplet was the most common in tandem triplet repeats. In contrast, glutamic acid was encoded by the most common repeat in *P. infestans*: (AAG)_n _. Like glutamine, this is also one of the most common tandemly repeated amino acids in the aforementioned organisms. Katti *et al*. [59] analyzed all the protein sequences from the SWISS-PROT database for single amino acid repeats, tandem oligo-peptide repeats, and periodically conserved amino acids; the results showed that repeats of glutamine, serine, glutamic acid, glycine and alanine seem to be fairly well tolerated in many proteins. Four of these five amino acids were found in our analysis and have also been reported in recent studies of complete genome coding sequences [36]. Such triplet repeat patterns in ORFs of oomycetes and other organisms could reflect functional selection of amino acid reiterations in the encoded proteins. Whole genome analyses have shown that repeat stretches of small/hydrophilic amino acids are more frequent in proteins [59]. The expansion of codons encoding such amino acids might be better tolerated than the expansion of hydrophobic amino acid stretches because they probably would not change the three-dimensional protein conformation as drastically. Therefore, nucleotide composition might strongly affect the structures and functions of encoded proteins, and it could be a determining force in the selection of SSRs in coding sequences.

We also explored the possibility that valuable information for mapping and for diversity or population structure studies could be obtained from these data. For this reason, microsatellite markers were evaluated for *in silico *cross-transferability among all three species. Many studies on organisms from different kingdoms have led to the development of markers amplifiable across species [34,44,54], even among *Phytophthora *species [17]. To our knowledge, this possibility has never been assessed *in silico *for any organism (including oomycetes). SSRs designed from EST/transcripts sequences are especially valuable owing to their genome location, which implies constraints on length, motif, abundance and flanking regions. The last of these is of particular interest for characterizing interspecies genetic diversity using common primers. Therefore, we designed primers for all SSR sets observed by species and identified those that were potentially cross-transferable. This approach was similar to a virtual PCR and provided candidate SSRs for experimental screening. The results revealed a small proportion of primers that could theoretically be used as transferable molecular markers (using a similarity criterion of 100%). This was an unexpected result because EST/transcript types of sequence are highly conserved and there is a close phylogenetic relationship among the *Phytophthora *spp. [27]. However, if the similarity criterion for alignment was less stringent (90%), 653 primer pairs were virtually transferable between at least two species. This number represents a remarkably large, diverse reservoir of markers that is potentially useful for diversity and population studies. With far fewer primers, an acceptable level of polymorphism has been found in previous studies of different organisms, where the primers are used to amplify a DNA region that has undergone SSR expansion in one lineage but not in related ones [8,54,60]. The transferability results might suggest that the organization of oomycete genomes shows marked variations. However, a more plausible explanation could be that the evolutionary distance between the selected taxa is greater than in other systems or organisms in which SSRs have been shown to be transferable (e.g. plants). Experimental validation of these hypothetically transferable SSRs and their polymorphism is on-going. This will verify the potential effectiveness of this *in silico *tool for finding transferable molecular markers for evaluating intra- and inter-specific diversity, instead of spending resources to validate them.

Evolutionary forces keep pathogen lineages and their hosts in an arms race, as each evolves new strategies for attack and defense [61]. The success of this arms race over time depends on the mechanisms by which pathogenicity factors evolve. Microsatellite mutation rates, ranging from 10^-6 ^to 10^-2 ^per generation, are higher than base substitution rates [10], and it is thus reasonable to assume that their presence in pathogenicity factors may contribute to variability in the relevant sequences [62]. In this study, we surveyed SSR abundance and distribution in putative pathogenicity factor sequences. The results were compared with SSR abundance and distribution in ribosomal and housekeeping sequences, which are presumed to be less variable because their fundamental functions in the organism are conserved. However, our results did not show higher SSR abundance in the pathogenicity factors. Differences from reference sequences (ribosomal and housekeeping) were not statistically significant (P > 0.05). This suggests that SSRs probably do not make a clear contribution to functional changes in pathogenicity factors, which allow *P. infestans, P. sojae *and *P. ramorum *to cause disease, but our study does not rule out the possibility that SSR polymorphisms may be present in pathogenicity genes.

## Conclusion

Databases for *P. sojae, P. infestans and P. ramorum *were inexpensive sources of SSRs. *In silico *investigation of SSRs is a significant step towards understanding the biological functions and nature of these important portions of the DNA. SSR markers are potentially useful tools for identifying *Phytophthora *species and for assessing genetic variation among populations. Evaluation of intraspecific genetic variation in *Phytophthora *concomitant with equivalent studies of host genetic variation could allow the relationships between host genotype variability, *Phytophthora *genotype and the selection pressures acting between them to be established. An important direct application of this study is as a resource for future population research.

## Methods

### Sources of ESTs and annotated transcript sequences

EST sequences from *P. infestans *(81974) were downloaded from the Phytophthora Functional Genomics Database [31]. The annotated transcript sequences available for *P. sojae *(19276) and *P. ramorum *(16066) were downloaded from the Department of Energy's Joint Genome Institute [32]. These redundant ESTs and transcript sequence sets were obtained in FASTA format. In addition, we downloaded 136, 318, and 171 sequences corresponding to putative or validated pathogenicity factors from *P. infestans *(National Center for Biotechnology Information [63]), *P. sojae *(Virginia Bioinformatics Institute [64]) and *P. ramorum *(DOE Joint Genome Institute [32]). From the same databases, ribosomal and housekeeping gene sequences were obtained for each of the organisms studied.

### Masking and assembly of the ESTs sequences from *P. infestans*

UniVec build # 3.2 was obtained from the NCBI web site FTP directory [65] and the latest repetitions database RepBase [33] EST were masked using the cross-match utility. PHRAP version 0.990329 was used to cluster the ESTs and to generate consensus sequences. Parameters phrap-miniscore 100 -minmatch 50 were used to generate consensus. The total consensus sequence count was 25965.

### Scanning of non-redundant EST and transcript sequences from *P. infestans*, *P. sojae *and *P. ramorum *for SSR survey

Non-redundant EST and transcript sequences were scanned using a local version of the Microsatellite Identification Tool (MISA) available from the Plant Genome Resources Center (PGRC) [66]. This program searches for both perfect and compound SSRs with 2 to 6 nucleotides in the basic repeat unit. It records repeat numbers and SSR locations inside the EST sequence and deposits these results in an output file. SSR redundancy was minimized by counting only a single match when there was more than one record for the same SSR locus. Minimum SSR length was determined by the number of repeats, which were (2/6) (3/4) (4/3) (5/3) (6/3); the first digit refers to the SSR repeat type and the second to the minimum number of repeat units (Table 1). For example, (2/6) means that a motif consisting of 2 bp and should have at least 6 repeats to be considered a microsatellite in our analysis. Pathogenicity factors, ribosomal genes and housekeeping genes were also scanned for SSRs using the same parameters (Table 6).

Total SSR numbers were normalized by calculating relative abundance. This was calculated as the number of SSRs per Mb of sequence analyzed so that the three sequence sets of different sizes could be compared. The relative density (bp/Mb) of each set of ESTs sequences was calculated by dividing the number of base pairs in the sequences (bp) contributed by each SSR by the total length of sequences examined (Mb); see Figures 1 and 2

### Primer design and cross-transferability

P3_in.pl and P3_out.pl perl scripts, which complement the MISA program, were adapted and used for automated selection and transfer of SSR-containing sequences from the database to the Primer3 program. Parameters used for the Primer3 program were as follows: optimal Tm of 57°C with a minimum and maximum of 50°C and 64°C respectively, and 50–75% GC content. The probability of dimer or hair-loop formation was low. The size of the PCR product is expected to be between 50 and 300 bp and no secondary structure.

To search for cross-transferability between paired *P. infestans-P. sojae*, *P. infestans- P. ramorum *and *P. ramorum- P. sojae*, each designed primer pair from each species was compared against all the SSR-containing sequences from the other species. The EMBOSS 3.0 Primersearch utility was used for this purpose. Cross-transferability among the three species was assessed manually.

In order to track all the data generated during this research, a MySQL relational database was designed and implemented. This database can be accessed at the URL listed in the references section [30]

### Statistical analysis

A proportion test was performed to evaluate differences between variables [67]. All statistical tests were performed using Statistix 8.0.

## Abbreviations

EST: Expressed Sequence Tag. SSR: Simple Sequence Repeat. EST-SSR: EST-derived SSR.

## Authors' contributions

DPG performed the data analysis and wrote the manuscript. AMP carried out all the programming activities and designed the database. LMQ, AJB and EB contributed to the conception and design of the study. AJB contributed to the writing of the manuscript. SR, EB and NJG directed and oriented the project and revised the manuscript. All authors read and approved the final manuscript.

## Figures and Tables

**Table 1:** Number and distribution of SSRs in consensus EST and transcript sequences.

	Organism		
	
	*P. infestans*	*P. sojae*	*P. ramorum*
Total number of sequences examined	25965	19276	16066
Total size covered by examined sequences (Mb)	12.573	29.249	22.097
Total number of SSRs identified	1660	5938	2838
Perfect ^a^	1466 (88.31 %)	4859 (81.83 %)	2554 (89.99 %)
Within a compound formation ^b^	194 (11.69 %)	1079 (18.17%)	284 (10.01 %)
Number of SSR-containing sequences:	1466 (5.65 %)	4265 (22.13 %)	2320 (14.44 %)
Number of sequences containing more than one SSR	152 (0.59 %)	1142 (5.92 %)	377 (2.35 %)
Total relative abundance (SSRs/Mb)	132.02	203.01	128.43
Total relative density (bp/Mb)	1803.66	2819.08	1723.24

Analysis was based on the MISA script, which identified di- to hexa-nucleotide repeat motifs (perfect and compound microsatellites) that are at least 12 bases in length.

^a ^*Perfect microsatellites *consist of a single repeat motif and are not interrupted anywhere by a base that does not fit the repeat structure

^b ^*Compound microsatellites *consist of two or more adjacent microsatellites with different repeat types

**Table 2:** Percentage, relative abundance and relative density of SSRs in the *P. infestans, P. sojae*, and *P. ramorum *sequence sets.

	**Motif length**	**Count**	**Percentage**	**Relative abundance**	**Relative density**
***P. infestans***	**di**	136	8.19	10.82	165.91
	**tri**	1009	60.78	80.25	1054.66
	**tetra**	335	20.18	26.64	328.01
	**penta**	64	3.86	5.09	77.55
	**hexa**	116	6.99	9.23	177.53

***P. sojae***	**di**	78	1.31	2.67	34.60
	**tri**	4863	81.90	166.26	2214.72
	**tetra**	434	7.31	14.84	187.22
	**penta**	88	1.48	3.01	47.35
	**hexa**	475	8.00	16.24	335.19

***P. ramorum***	**di**	28	0.99	1.27	15.39
	**tri**	2358	83.09	106.71	1377.36
	**tetra**	199	7.01	9.01	108.79
	**penta**	25	0.88	1.13	16.97
	**hexa**	228	8.03	10.32	204.74

^a ^Percentage was calculated for each organism on the basis of the corresponding total SSRs count

^b ^Relative abundance is defined as the total number of SSR s per Mb of sequence analyzed

^c ^Relative density is defined as a the total sequence length (bp) contributed by each SSR per Mb of DNA of total sequence analyzed

**Table 3:** Most common repeat motifs identified from perfect and compound microsatellites in the three oomycetes analyzed.

***P. infestans***			***P. sojae***			***P. ramorum***		
**Motif **^**a**^	**Count**	**Percentage**	**Motif**	**Count**	**Percentage**	**Motif**	**Count**	**Percentage**

AAG/CTT	258	15.54	AGC/CGT	1318	22.20	AGC/CGT	630	22.20
ACG/CTG	158	9.52	ACG/CTG	964	16.23	ACG/CTG	527	18.57
AGC/CGT	128	7.71	AAG/CTT	833	14.03	AAG/CTT	383	13.50
ACC/GGT	114	6.87	AGG/CCT	710	11.96	AGG/CCT	315	11.10
AGG/CCT	86	5.18	CCG/CGG	554	9.33	CCG/CGG	220	7.75
AAC/GTT	68	4.10	ACC/GGT	215	3.62	ACC/GGT	131	4.62
CCG/CGG	67	4.04	AAC/GTT	134	2.26	AAC/GTT	74	2.61
AC/GT	55	3.31	ACT/ATG	99	1.67	ACT/ATG	64	2.26
AG/CT	54	3.25	AGCG/CGCT	70	1.18	AGCG/CGCT	49	1.73
ACT/ATG	53	3.19	CG/CG	46	0.77	ACGC/CGTG	27	0.95
AAT/ATT	52	3.13	AGGC/CCGT	33	0.56	CG/CG	23	0.81
AAAC/GTTT	50	3.01	AGT/ATC	32	0.54	ACCG/CTGG	23	0.81
AAAT/ATTT	36	2.17	ACCG/CTGG	29	0.49	ACCACG/CTGGTG	15	0.53
AGT/ATC	25	1.51	AAGG/CCTT	28	0.47	AGT/ATC	14	0.49
AT/AT	23	1.39	ACGG/CCTG	28	0.47	AGCC/CGGT	14	0.49

^a ^The same constraints on minimum length cited for Table 1 were used.

**Table 4:** The longest SSR motifs found in EST consensus and transcript sequences for the three oomycetes analyzed.

	**Repeat Type **^**a**^				
	
	**di**	**tri**	**tetra**	**penta**	**Hexa**
***P. infestans***	TA (28)	ATT (69)	CAAG (20)	ATTTT (20)	CCTGCA (36)
	TG (28)	ATT (33)	AAAT (20)	AAAGA (20)	GTTGAG (30)
	AC (24)	AAG (30)	ATTA (20)	CGTGG (15)	CAGCAA (24)
	AT (24)	AAC (27)	ATAA (16)	TTGTT (15)	AGCAGG (24)

***P. sojae***	AG (28)	AAG (66)	AAGA (32)	CAAGC (30)	TCGGCA (144)
	AG (28)	AGG (57)	GCCT (24)	GTGTA (25)	TCTACT (96)
	CT (18)	AAG (51)	GCCT (24)	TCTCA (20)	GCTACG (54)
	CT (18)	AGG (48)	AGCC (24)	AGAGC (20)	AAGACC (54)

***P. ramorum***	AG (14)	AGC (45)	CTGG (16)	CAAAA (15)	CAGGAG (90)
	CG (14)	CGT (30)	CTTC (16)	AGCGC (15)	AGCGAC (90)
	AG (12)	AAG (30)	CAGC (16)	CAAGC (15)	GAAGAC (48)
	AC (12)	AGG (30)	GTGC (16)	CCAAG (15)	CGAGTC (42)

^a ^Canonical notation was used for all SSRs

**Table 5:** Distribution of SSRs in three functional categories of genes

		Sequences examined	Number of identified SSRs	SSR-containing sequences (%)
	
***P. infestans***	Pathogenicity factors^a^	136	24	16.91%
	Ribosomal genes	44	2	5.54%
	Housekeeping genes^b^	41	6	14%
	
***P. sojae***	Pathogenicity factors^a^	318	50	11.63%
	Ribosomal genes	52	11	17.30%
	Housekeeping genes^b^	22	3	13.63%
	
***P. ramorum***	Pathogenicity factors^a^	171	16	9.35%
	Ribosomal genes	50	3	6%
	Housekeeping genes^b^	48	5	8.33%

^a^Putative sequences of cutinases glucanses, polygalacturonases, pectatoliases, cellulases, elicitins and proteins with conserved RXLR motif were selected for this analysis

^b^Putative gene sequences of actin, cytochrome P450-like protein and NADH hydrogenase were selected for this analysis.

**Table 6:** *In silico *determination of potential cross-transferability of the SSRs between *Phytophthora *species

	Primer transferability ^a^		
	
	100%	95–99 %	90–94 %
*P. infestans *– *P. sojae*	7	21	91
*P. infestans *– *P. ramorum*	6	8	50
*P. sojae *– *P. ramorum*	27	126	282
*P. infestans *– *P. sojae *– *P. ramorum*	4	19	61

^a ^When an EST or transcript sequence matched primer pairs designed from species 1 and species 2, they were counted as one primer pair.
